# The Influence of the Ozonation Process on the Quality Parameters and Physicochemical Stability of Horse Meat

**DOI:** 10.3390/molecules31183315

**Published:** 2026-09-18

**Authors:** Renata Stanisławczyk, Tomasz Piechowiak, Marian Gil, Dorota Grabek-Lejko, Maciej Balawejder

**Affiliations:** 1 Department of Agricultural Processing and Commodity Science, Institute of Food and Nutrition Technology, Faculty of Technology and Life Sciences, University of Rzeszow, Zelwerowicza 4, 35-601 Rzeszow, Poland; mgil@ur.edu.pl; 2 Department of Chemistry and Food Toxicology, Institute of Food and Nutrition Technology, Faculty of Technology and Life Sciences, University of Rzeszow, 1a Ćwiklińskiej St., 35-601 Rzeszow, Poland; tpiechowiak@ur.edu.pl (T.P.); mbalawejder@ur.edu.pl (M.B.); 3 Department of Bioenergetics, Food Analysis and Microbiology, Institute of Food and Nutrition Technology, Faculty of Technology and Life Sciences, University of Rzeszow, Zelwerowicza 4, 35-601 Rzeszow, Poland; dgrabek@ur.edu.pl

**Keywords:** horse meat, ozone, oxidation, fatty acid, texture parameters, sensory quality, volatile compounds profile

## Abstract

The aim of this study was to demonstrate the effect of gaseous ozonation at a concentration of 10 ppm on the quality of horse meat. Samples of the *longissimus thoracis* (LT) muscle were exposed to ozone for 1, 5, and 15 min and then stored at 4 °C for 6 and 48 h. Ozonation caused a deterioration in the water-holding capacity of muscle tissue. Significant changes in the color of horse meat were observed, as longer exposure to ozone intensified oxidative processes in the meat. The volatile compound profile after 48 h indicated significant lipid degradation and an increase in aldehydes, typical of fat rancidity. Texture analysis showed that short ozonation increased meat tenderness, while 15 min exposure led to a deterioration of the texture characteristics of horsemeat. Sensory evaluation confirmed a decrease in the tenderness and desirability in horse meat samples after prolonged ozonation. Ozone demonstrated high antimicrobial efficacy, particularly after 15 min of exposure, where yeast and mold counts reached their lowest levels at 3.24 and 3.04 log_10_ CFU/mL, respectively, across both time intervals. After 48 h of storage, a 15 min ozone treatment effectively minimized total bacterial contamination to 3.96 log_10_ CFU/mL. The study results indicate that ozone can extend the microbiological shelf life of horse meat, but its application parameters must be precisely selected to limit adverse oxidative and qualitative changes.

## 1. Introduction

Although horse meat represents a niche segment of the global meat sector, worldwide production has remained relatively stable in recent years, reaching 775,543 tonnes in 2022. In the European Union, horse meat production amounted to approximately 44,000 tonnes, with Spain, Italy, and Poland among the principal producing countries. EU market supply was additionally supplemented by more than 16,000 tonnes of imports, predominantly from South American countries [1]. Horse meat availability for human consumption varies considerably among regions. According to the 2023 FAOSTAT Food Balances, the global average per capita supply of horse meat available for human consumption was approximately 0.10 kg/year. Substantially higher values were reported for Mongolia (11.97 kg/capita/year), Kazakhstan (8.38 kg/capita/year), and Kyrgyzstan (4.40 kg/capita/year). In Europe, the corresponding supply was markedly lower, amounting to 0.42 kg/capita/year in both Belgium and Italy and 0.28 kg/capita/year in Switzerland [2,3]. These figures indicate that, although horse meat contributes only marginally to global meat supply, its availability remains relevant in selected Asian and European markets with established culinary traditions [3]. Horse meat is characterized by high nutritional value and is an excellent source of nutrients and minerals. This raw material contains approximately 75% water, approximately 20% protein, and approximately 1% mineral salts. Muscle fat ranges from 0.7% to 4.5%, while minerals range from 0.8% to 1.8%. The high nutritional value of meat—resulting from its high protein, iron, and vitamin content—makes it a perishable product [4,5]. Susceptibility to rapid microbial proliferation significantly shortens its shelf life, posing a challenge during storage [6,7].

An increasing number of consumers are paying attention to food quality and safety. Ensuring food safety during production while maintaining high nutritional value and fresh appearance requires the use of unconventional technologies other than thermal ones. These include oxidation technologies such as ozonation, cold plasma, or ionization. The principle of operation of these techniques allows for maintaining food safety and extending the shelf life of products while maintaining the highest possible food quality [8]. The use of ozone (O_3_) is becoming an increasingly popular method of cleaning and disinfection, mainly due to its effectiveness and safety for manufactured products and human health [9]. Ozone is a strong oxidizing agent that effectively reduces microbiological contamination, thus extending the shelf life of food products. Previous research results show that bacteria have varying resistance to ozone. Previous studies have shown that microorganisms differ in their susceptibility to ozone. Under appropriate treatment conditions, ozone can exhibit antimicrobial activity against both Gram-positive bacteria, including *Listeria monocytogenes*, *Staphylococcus aureus*, and *Enterococcus faecalis*, and Gram-negative bacteria, such as *Yersinia enterocolitica*, *Pseudomonas aeruginosa*, *Salmonella* Typhimurium, and *Escherichia coli*. Ozone may also inactivate viruses, filamentous fungi, yeasts, including *Candida parapsilosis* and *Candida tropicalis*, as well as fungal and bacterial spores [10,11,12]. The effectiveness of ozone disinfection depends on the organism and is also dependent on other factors, e.g., environmental conditions, substrate (material) properties and operational properties (concentration, type of treatment, temperature) [13]. Ozone can be applied to food products in the gas phase or dissolved in the aqueous phase [14,15,16]. The undoubted advantage of using ozone is the fact that excess ozone is rapidly and automatically decomposed, producing oxygen. In the presence of food products, it decomposes very quickly, leaving no residue [12,17,18].

The use of ozone in meat processing may modify physicochemical and nutritional parameters, as well as sensory characteristics, with the most pronounced changes generally observed in surface color. The effects of ozonation depend on the type of raw material. In red meat, ozone treatment may promote tissue oxidation, resulting in undesirable color changes, deterioration of overall quality, and accelerated lipid oxidation. The effects of ozone on physicochemical properties depend on the treatment medium (gaseous or aqueous), ozone dose, exposure time, temperature, and the specific characteristics of the meat. As strong oxidants, ozone and other reactive oxygen species (ROS) catalyze the conversion of myoglobin to metmyoglobin [19,20]. This process leads to unfavorable visual changes and discoloration of the meat, which is consequently manifested by a reduction in the CIE a* parameter value [21].

It is worth noting that the existing literature has mainly focused on the effect of ozonation on commonly consumed types of meat, including poultry [22,23,24], beef [15,25,26,27], pork [28], and fish [29,30,31], with particular emphasis on improving microbiological safety. However, comprehensive studies evaluating the effects of ozone on horse meat are still lacking. Horse meat may be particularly susceptible to ozone-induced oxidative changes because of its relatively high proportion of polyunsaturated fatty acids and its high myoglobin content compared with beef or pork. The presence of these oxidation-sensitive constituents may increase the risk of lipid oxidation and myoglobin oxidation, potentially leading to deterioration of color, flavor, and overall product quality during ozonation [4,5]. Therefore, this study aims to address this research gap by comprehensively evaluating the effects of ozonation on the microbiological quality, chemical composition, physicochemical and sensory properties, and texture parameters of horse meat.

## 2. Results and Discussion

### 2.1. Chemical Composition

Currently, the literature does not provide specific and comprehensive data on the effect of ozonation on the chemical composition of horse meat. Studies conducted by Fathul Karamah and Wajdi [32] on poultry meat subjected to ozonation for 40, 80, and 120 min at temperatures of 3, 26, and 37 °C and ozone concentrations of 0.21 and 0.38 mg/L indicate a slight decrease in water content in the analyzed raw material by 0.6%, 0.6%, and 1%. The corresponding reductions in protein content were 0.17, 0.5, and 0.3 percentage points, respectively. According to the authors, this distribution of results indicates that ozone has no significant effect on the content of the specified components in the analyzed raw material.

Table 1 presents data on the chemical composition of ozonated horse meat. These data indicate that both ozonation time and the duration of refrigerated storage following ozonation significantly influenced the water and fat content of horse meat (*p* < 0.05). The protein content in the horse meat samples did not change significantly as a result of the ozonation process. After 48 h of refrigerated storage, horse meat samples ozonated for 1 and 5 min exhibited lower fat content compared to the control sample. Horse meat samples subjected to 1 min of ozonation also showed a significant decrease in water content compared to the control sample within the analyzed timeframe. After 6 h of storage after ozonation, a significantly lower fat content was found in horse meat samples ozonated for 5 min compared to the control sample. Extending the storage time from 6 to 48 h after ozonation resulted in a significant reduction in fat content—from 6.60% in control samples to 5.17% and 5.03% in samples ozonated for 1 and 5 min, respectively. It can be assumed that the water content of meat after ozonation decreases primarily due to changes in protein structure, particularly protein denaturation, which reduce the ability of proteins to retain water within the tissue. This results in an increase in cooking loss and forced drip loss.

### 2.2. Hydration Properties

The data in Table 2 concern the hydration properties of ozonated horse meat. These data indicate that both ozonation time and cold storage significantly increase the cooking loss and forced drip loss of horse meat. Control samples of horse meat, not subjected to ozonation, had the greatest capacity to retain water within the muscle structure. Ozonation of horse meat for 1, 5, and 15 min led to a significant increase in cooking loss and forced drip loss compared to the control samples of horse meat. Cold storage of horse meat samples after ozonation from 6 to 48 h also led to a deterioration in the analyzed hydration properties. Such changes were expected, as ozonation of meat causes structural changes in muscle proteins induced by strong oxidation. This reduces the proteins’ ability to bind and retain water. Ozone, a strong oxidant, affects myofibrillar proteins, primarily myosin, leading to their degradation, fragmentation, and the formation of new cross-links. These changes denature proteins and reduce their ability to retain water within the muscle tissue structure [33]. Ozonation also causes proteins to become more hydrophobic, limiting their interaction with water. Due to the damage to the protein structure (denaturation) by ozone, meat retains water less effectively under external forces, resulting in greater forced leakage. As a result, the water bound by the proteins is released. During heat treatment, ozone-denatured proteins contract more intensely, which “squeezes” more water from the muscle tissue. This causes higher cooking loss, resulting in decreased meat juiciness.

### 2.3. pH Values

Measurements of the pH of horse meat (Table 2) show that both the duration of ozonation and the length of cold storage after ozonation, as well as the interaction effect between ozonation time and cold storage length, had a statistically significant effect on the value of the analyzed parameter (*p* < 0.05). After 6 h of refrigerated storage, a significant increase in pH value was observed in horse meat samples ozonated for 5 min compared with the control samples. Significantly higher pH values, ranging from 5.94 to 5.98, were observed in the horse meat samples after 48 h of cold storage after ozonation. This distribution of results indicates that the acidity of horse meat was influenced more by the cold storage time after ozonation than by the duration of the ozonation process. The obtained distribution of results was undoubtedly influenced by the enzymatic and microbiological protein degradation occurring during cold storage of the meat. Alkaline nitrogen compounds formed during storage at low temperatures cause an increase in the pH value, which in turn reduces the acidity of the meat. Fathul Karamah and Wajdi [32], examining the effect of ozone concentration on the pH of poultry meat, showed that higher ozone concentrations (0.38 mg/L) lowered the pH of chicken meat by 0.01, while lower ozone concentrations (0.21 mg/L) lowered the pH by 0.06. Taking into account the process duration of 40, 80, and 120 min, it resulted in a decrease in the pH value by approximately 0.01–0.02. The authors’ research results indicate that ozonation had no effect on the pH value of poultry meat. Trindade and Kushida [34] also found no change in the pH value in the case of poultry carcasses washed with water at a concentration of 1.5 mg/L for 45 min.

### 2.4. Color Parameters and Pigment Levels

Table 3 presents the lightness (L*) and color parameters of horse meat as affected by ozonation and subsequent refrigerated storage. Ozonation time and refrigerated storage duration significantly affected the color parameters of horse meat, whereas the total pigment content was significantly affected by ozonation time, storage duration, and the interaction between these factors (*p* < 0.05).

Extending the exposure time of horse meat samples to ozone (1 → 5 → 15 min), both after 6 and 48 h of refrigerated storage after ozonation, led to an increase in the L* component and the yellow b* color value, and a decrease in the red a* color value of the horse meat samples. A significant increase in the lightness of the horse meat samples and a significant increase in the b* color parameter were demonstrated in both time intervals between the samples ozonated for 15 min and the other experimental groups (*p* < 0.05). Considering the overall level of pigments in the horse meat samples, it should be concluded that the ozonation process led to a decrease in the level of pigments in the analyzed raw material. Significantly, the highest level of pigments was demonstrated in the control horse meat samples, both after 6 and 48 h of refrigerated storage (*p* < 0.05). Extending the exposure time of horse meat samples to 5 and 15 min resulted in a significant reduction in pigment concentration compared to the other experimental groups. This trend was observed after 6 and 48 h of refrigerated storage. The observed changes in instrumental color were accompanied by a decrease in myoglobin and oxymyoglobin levels and an increase in metmyoglobin, supporting the interpretation that ozonation promoted pigment oxidation. The increased proportion of MMb may explain the reduction in redness and the development of a brownish-gray color in the ozonated horse meat samples [19,21,33]. Changes in lightness and yellowness may additionally have been associated with ozone-induced modifications of surface proteins, surface moisture, or heme pigments, as suggested in previous studies on ozonated meat [25,26,33]. However, these mechanisms were not directly assessed in the present study and are therefore presented only as possible explanations. Overall, the measured changes in myoglobin forms and CIE color parameters indicate that ozonation contributed to pigment oxidation and color deterioration. This prevents rebinding of oxygen, even if the meat is later exposed to air. An attempt to determine the effect of ozone on the color parameters of chicken breast meat was undertaken by Cho et al. [35]. The cited authors demonstrated that the use of ozone at a concentration of 10 mg O_3_/h resulted in a decrease in the L* and a* values and an increase in the b* color parameter during 2–3 days of storage (*p* < 0.05). This relationship is also confirmed by other studies. Ayranci et al. [24] exposed turkey breast meat to gaseous ozone at a concentration of 10 g/m^3^ and showed, after 8 h of treatment at 22 °C, an increase in the lightness of the meat color L* from 34.43 to 41.97 and a decrease in the value of the red color component a* from 2.08 to 0.35. Other studies [22] showed that subjecting poultry meat samples to gaseous ozone treatment resulted in a significant change in the parameters L*, a* and b*. The most desirable color change was observed after the third day of storage at 4 °C in samples exposed to ozone at a concentration of 10 ppm for 15 min.

### 2.5. Thiol Group Content in Soluble Meat Proteins

**Figure 1:**
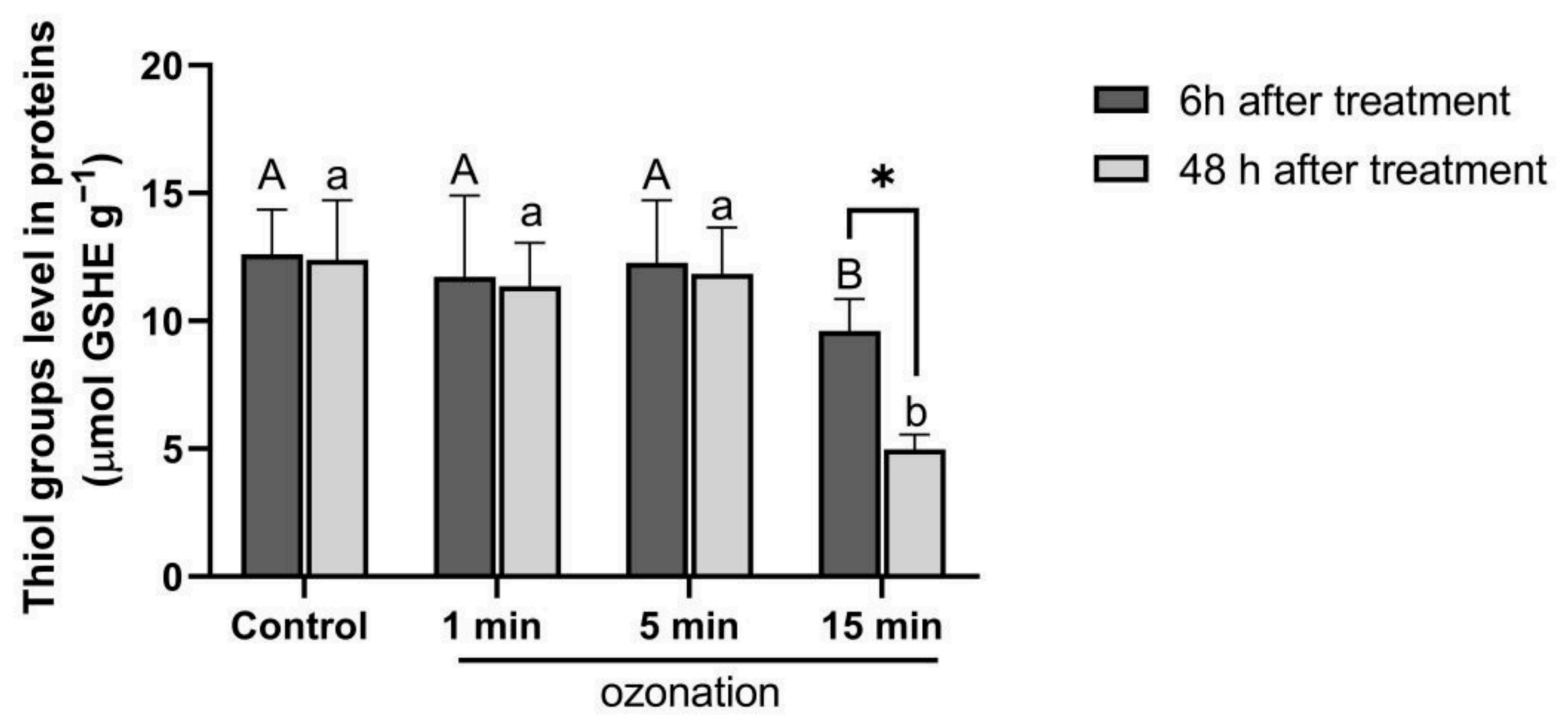
The thiol group content in soluble meat proteins. Mean values ($\bar{x}$ ± SD, *n* = 6). marked with the same lowercase or uppercase letter are not statistically significant in relation to each other for the corresponding storage time according to Tukey’s test at α = 0.05. Mean values marked with * are statistically significant relative to each other according to Tukey’s test.

Figure 1 shows the content of thiol groups in soluble meat proteins. The literature provides information [33] that with prolonged exposure, the strong oxidizing properties of ozone begin to dominate, resulting in excessive oxidation of sulfhydryl (-SH) groups and the formation of disulfide bridges. Proteins begin to form dense aggregates (cross-linking), which causes fiber hardening. Furthermore, with prolonged exposure, ozone can cause proteins to lose their water-binding capacity (juice leakage), making the meat drier and firmer.

Furthermore, the decrease in the content of -SH groups is directly correlated with the increase in hardness, chewiness, and elasticity, which is reflected in the research results in this study (Table 4). The highest content of thiol groups in horse meat proteins was demonstrated in control samples after 6 h (12.61 µmol/g) and 48 h (12.39 µmol/g) of storage at low temperatures after the ozonation process. Ozonation of horse meat samples for 15 min resulted in a significant decrease in the content of thiol groups in both time intervals compared to the other experimental groups (*p* < 0.05). 15 min contact with ozone reduced the content of thiol groups to the significantly lowest level, respectively: 9.60 after 6 h of storage and 4.98 µmol/g after 48 h of cold storage. These results indicate rapid oxidative changes in the meat.

### 2.6. Level of Lipid Peroxidation (TBARS)

**Figure 2:**
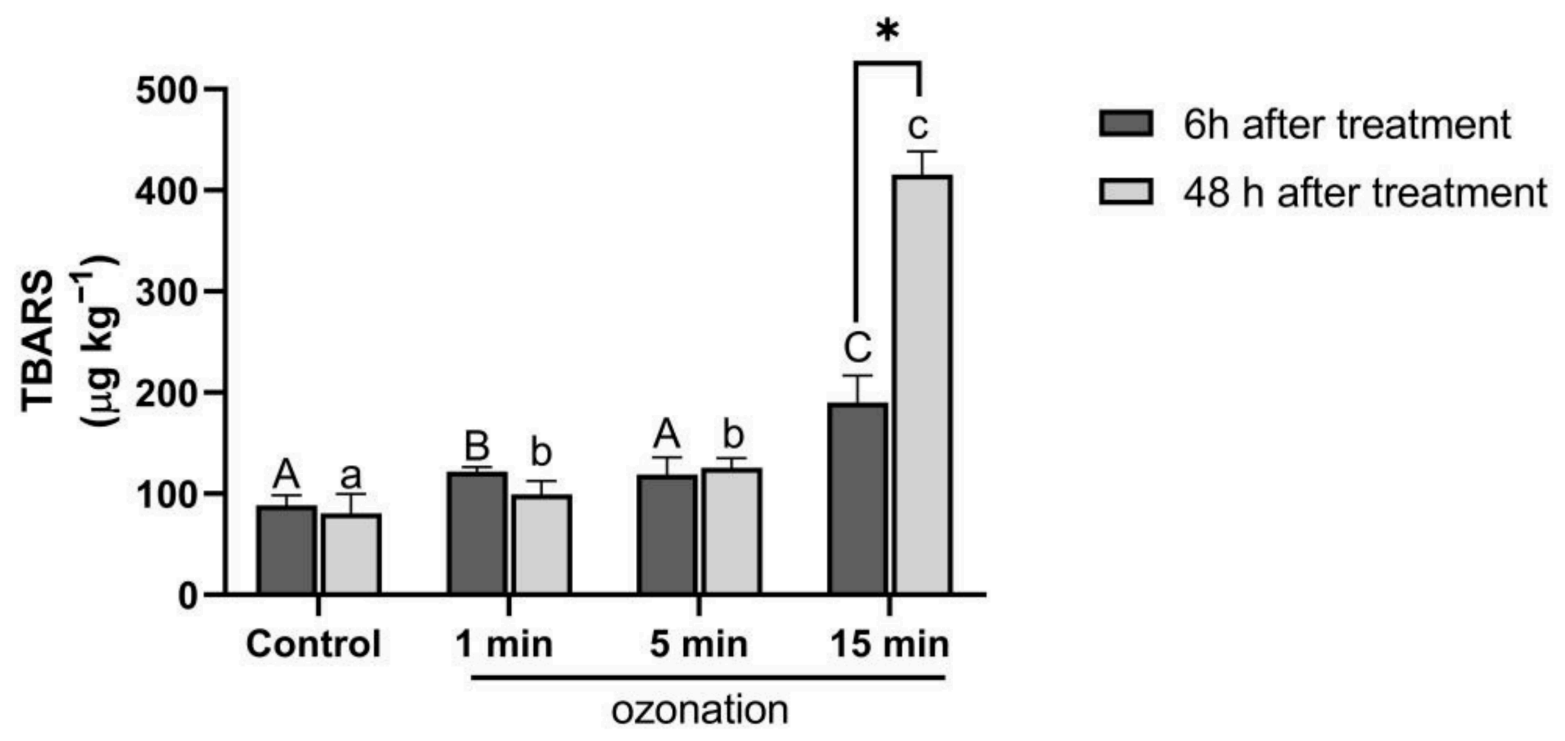
The level of lipid peroxidation in meat expressed as the content of substances reacting with thiobarbituric acid (TBARS). Mean values ($\bar{x}$ ± SD, *n* = 6) marked with the same lowercase or uppercase letter are not statistically significant in relation to each other for the corresponding storage time according to Tukey’s test at α = 0.05. Mean values marked with * are statistically significant relative to each other according to Tukey’s test.

The decrease in thiol groups in muscle tissue is often accompanied by lipid oxidation. The research results obtained in this study confirm this relationship. Figure 2 presents the level of lipid peroxidation in meat, expressed as the content of substances reactive with thiobarbituric acid. The demonstrated increase in the content of thiobarbituric acid-reactive substances (TBARS) is direct evidence of progressive oxidative rancidity under the influence of ozone. The lowest TBARS values were observed in the control samples, both after 6 and 48 h of refrigerated storage, at 88.35 and 80.71 µg MDA/kg, respectively.

A significant increase in the TBARS value was obtained for horse meat samples ozonated for 15 min compared to the other experimental groups (*p* < 0.05). The obtained distribution of results confirms the fact that longer ozonation (15 min) intensifies lipid peroxidation. Extending the storage time from 6 to 48 h significantly increased the TBARS values (*p* < 0.05), indicating enhanced lipid peroxidation. The highest value, 415.74 µg MDA/kg, was recorded in samples ozonated for 15 min and subsequently stored for 48 h under refrigerated conditions. Scientific publications on the effect of ozone on the value of the analyzed index value emphasize that the ozonation process affects the increase in the TBARS index [24,36,37]. For example, in the studies by Cho et al. [26], treating ground Hanwoo meat with gaseous ozone (10 × 10^−6^ kg O_3_/h) at 4 °C for 3 days showed an increase in the TBARS value from 0.66 mg malonaldehyde/kg of meat on the first day to 0.79 mg malonaldehyde/kg of meat after three days of storage. It is noteworthy that in our study, the TBARS value did not exceed 1 mg malonaldehyde/kg of meat, which is the acceptable sensory threshold indicating the development of a rancid taste. The distribution of results obtained in this study was undoubtedly influenced by the fact that horse meat contains a large amount of unsaturated fatty acids [38,39,40], which makes this raw material more susceptible to oxidation processes. Moreover, there are reports in the literature indicating that increased ozone concentration may induce oxidation of some food compounds [41].

### 2.7. The Volatile Compounds Profile

The analysis of the volatile compound profile showed that ozonation markedly affected the composition of the volatile fraction of the meat, with the observed changes depending on both the ozone exposure time and storage period (Table 5). After 6 h, benzaldehyde (44.71%) was the predominant compound in the control sample, whereas it was not detected in the ozonated samples. At the same time, the proportion of Z,E-2,13-octadecadien-1-ol increased, reaching the highest value after 5 min of ozonation (69.53%). After 15 min, 9,17-octadecadienal (20.50%) was detected.

After 48 h of storage, further changes in the volatile compound profile were observed. The highest proportion of Z,E-2,13-octadecadien-1-ol was recorded after 1 min of ozonation (74.20%), whereas its proportion decreased markedly after 5 and 15 min. In these samples, the proportion of the second octadecadien-1-ol isomer increased, particularly after 15 min of ozonation (57.05%). This is further supported by the significant increase in the TBARS index, which increased with increasing ozone exposure time and reached the highest levels in horse meat samples subjected to 15 min of ozonation after 48 h of cold storage. Overall, these results indicate that ozonation modified the volatile compound profile of the meat, probably as a result of oxidative processes, with the extent of these changes depending on ozone exposure time and storage period.

### 2.8. Fatty Acids Composition

Table 5 presents data on the fatty acid content of horse meat samples 6 h after ozonation. These data indicate that oleic acid (C18:1) constitutes the largest component of the acid profile (approximately 38–41%). The fatty acid profile after 6 h of storage remains relatively stable, and differences between the control and ozonated samples are small. During the analyzed time period, the ozonation process did not significantly affect the content of saturated fatty acids (SFAs). The values were stable (24.713–25.431%). There were also no significant differences in the level of monounsaturated fatty acids (MUFA) (50.613–53.188%), indicating that ozone does not degrade MUFA at an early stage. A similar relationship was observed for polyunsaturated fatty acids (PUFA). The minimal variations observed (21.381–24.547%) indicate that the ozonation process, regardless of exposure time, does not significantly alter lipid structure and has not yet led to intensive oxidation of the horse meat samples. This is consistent with the data in Table 6, where oxidative processes are initiated only after a short storage period.

Much more dynamic changes were observed after 48 h of storage of horse meat samples after ozonation. The fatty acid profile observed during the analyzed time interval confirms the intensification of oxidative processes in horse meat. In samples subjected to longer ozonation (15 min), a significant decrease in the content of polyunsaturated fatty acids (PUFA), which are particularly susceptible to oxidation, was observed. Extending the exposure time of horse meat samples to 15 min resulted in a decrease in the content of polyunsaturated fatty acids (PUFA) to the lowest value of 18.23%. Simultaneously, the increase in the content of saturated fatty acids (SFAs) and the relative increase in the content of MUFA may result from the degradation of PUFA to products with a lower degree of unsaturation. After 15 min of ozonation, the content of saturated fatty acids (SFA) and monounsaturated fatty acids (MUFA) reached the highest levels: 28.38% and 53.38%, respectively. A detailed analysis of the data presented in Table 6 showed that the proportion of linoleic acid in horse meat decreased significantly to 14.76% after 15 min of ozonation. This reduction may indicate the susceptibility of linoleic acid to ozone-induced oxidation. In the analyzed time variant, 15 min of ozonation also resulted in an increase in the proportions of arachidonic acid (C20:4) and oleic acid to 2.23% and 41.92%, respectively. These data clearly indicate that longer ozonation times combined with meat storage contribute to the deterioration of fat stability, which may result in a loss of nutritional value.

### 2.9. Texture Parameters

Data regarding the texture parameters of horse meat are included in Table 4. These data indicate that the duration of ozonation, the length of cold storage after ozonation, and the interaction effect between ozonation time and cold storage length had a statistically significant effect on variation in shear force values for horse meat (*p* < 0.05). In this study, after both 6 and 48 h of cold storage, a significant decrease in shear force was noted between the control sample and the samples subjected to 1 min of ozonation. However, after 15 min of ozonation, the opposite trend was observed, i.e., in both time intervals, there was a significant increase in the shear force required to cut horse meat samples compared to the other experimental groups.

Analysis of the TPA results for ozonated horse meat also revealed the same relationship for hardness 1 and hardness 2. This indicates that the 15 min ozonation process of horse meat at both time intervals tested resulted in deterioration of the specified texture parameters. The deterioration of the analyzed texture parameters of horse meat can be explained by the fact that ozone, as an oxidant, could have caused cross-linking between protein chains, primarily myosin, after 15 min. This could have resulted in the formation of a stiff and dense protein network, offering greater resistance to cutting the meat samples. Ozone treatment may have induced changes in proteins at the meat surface, potentially leading to the formation of a harder surface layer or barrier that contributed to the increased overall hardness of the meat samples. However, because protein denaturation and structural changes across the sample depth were not directly assessed, this mechanism should be considered only a possible explanation. The decrease in the ability of proteins to bind water due to oxidation, as demonstrated in Table 2, undoubtedly influenced the obtained results regarding the texture of the horse meat samples. As a result of the loss of water from the interfiber spaces, the muscle fibers become closer together, becoming more compact and harder.

Considering the results in Table 4, it should be noted that the duration of ozonation, the length of cold storage after ozonation, and the interaction effect between ozonation time and cold storage length had a significant effect on stiffness 5 and stiffness 8, cohesion, resilience, and chewiness of the tested horse meat samples (*p* < 0.05). Furthermore, the duration of ozonation and the length of cold storage after ozonation significantly influenced the adhesiveness and elasticity of the tested horse meat samples (*p* < 0.05). It is noteworthy that after 5 min of ozonation, stiffness 5 and stiffness 8, cohesion, resilience, and elasticity reached the lowest values in the tested horse meat samples. This indicates that after 5 min of ozonation, protein fragmentation may dominate, resulting in “tender” meat (broken bonds between fibers). Such meat becomes more plastic and less elastic. However, after 15 min of longer exposure, the trend reverses in both time intervals, resulting in an increase in the analyzed texture parameters. The structure of the muscle tissue becomes compact and chemically stiffened. The meat surface becomes drier and less viscous. Ozone acts as a drying agent on the surface. This phenomenon is likely caused by strong oxidation, leading to intense protein cross-linking (the formation of new disulfide bridges). This results in the formation of a dense, stiff network. The muscle tissue becomes more mechanically elastic and responds more rapidly to the release of pressure, resulting in higher resilience. This is also reflected in the increased chewiness of the horse meat samples. The 15 min ozonation process led to significant aggregation; meat fragments may be more difficult to separate, which likely directly contributed to the increased chewiness parameter of the horse meat samples.

### 2.10. Sensory Properties

Table 7 presents data on the sensory quality parameters of ozonated horse meat. These data indicate that the duration of ozonation and the length of refrigerated storage after ozonation significantly influence the development of odor and flavor (desirability), juiciness, and tenderness in horse meat samples. In turn, the duration of the ozonation process significantly influences the intensity of odor and flavor of the analyzed raw material. Considering the exposure time of horse meat samples to ozone, it was demonstrated that extending the ozonation time to 15 min resulted in a significant deterioration in the tenderness of the meat in both time intervals. However, the juiciness of the horse meat samples was at the same level as the juiciness of the control samples. Horse meat samples received the lowest scores for the analyzed sensory quality parameters at the indicated time. Such changes were to be expected, as oxidized proteins lose their ability to bind water as a result of ozonation, which resulted in water loss from the horse meat samples and a deterioration in their hydration properties. As meat loses moisture, the fibers shrink and harden, becoming dry and stringy, which is perceived by consumers as a decrease in tenderness. These phenomena are confirmed by the results presented in this study (Table 1 and Table 2). It should be noted that horsemeat samples received high scores for odor and flavor intensity, ranging from 4.00 to 4.50 points, both after 6 and 48 h of refrigerated storage after ozonation. Horsemeat samples also received high scores for odor and flavor desirability after 6 h of refrigerated storage, not only after ozonation but also in the control samples. Meanwhile, after 48 h of ozonation, the analyzed sensory quality parameters of horsemeat ranged from 3.25 to 4.00 points for odor desirability and from 3.75 to 4.00 points for flavor desirability.

### 2.11. Microbial Analysis

**Figure 3:**
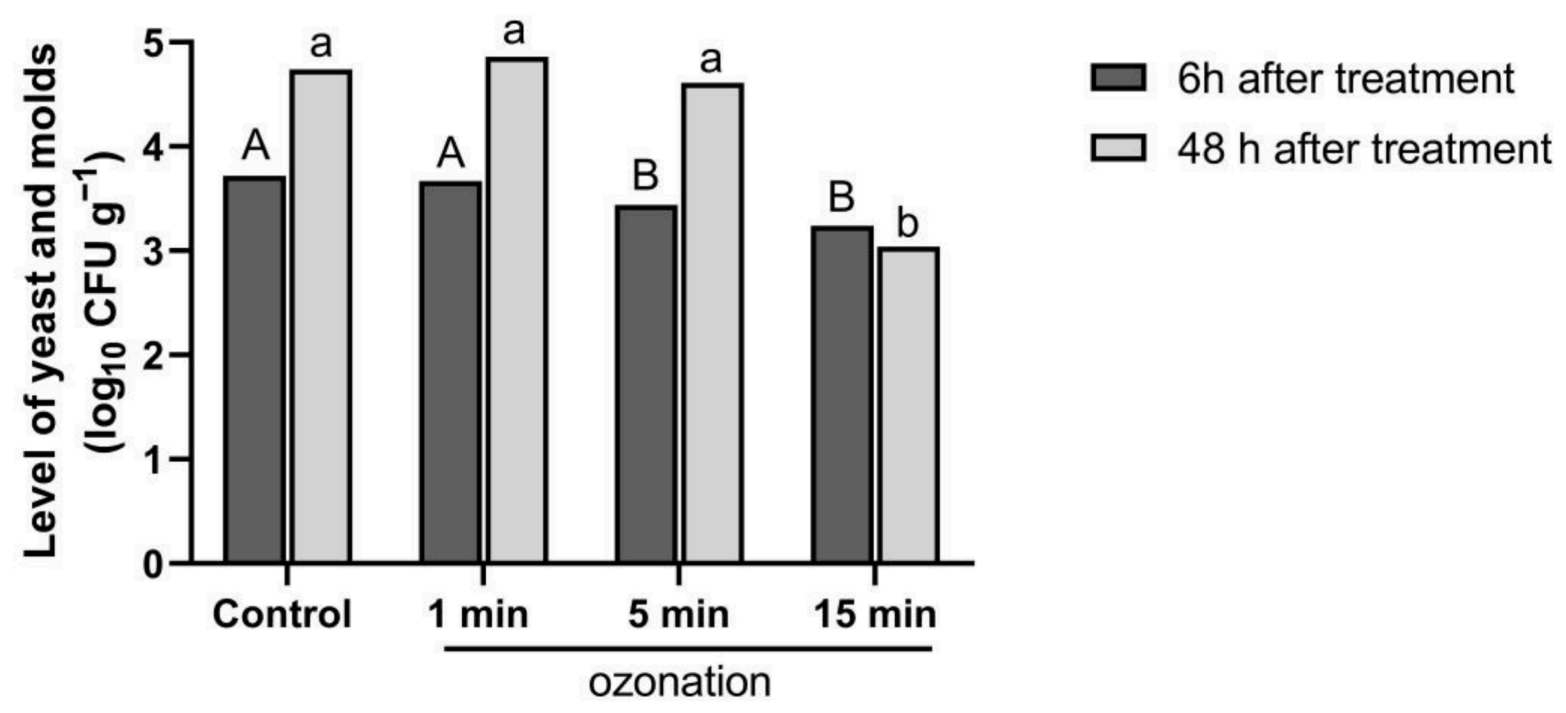
Levels of yeast and molds in ozonated and control (zero time) meat samples after 6 and 48 h of storage under cold conditions. Mean values (*n* = 6) marked with the same lowercase or uppercase letter are not statistically significant in relation to each other for the corresponding storage time according to Tukey’s test at α = 0.05.

**Figure 4:**
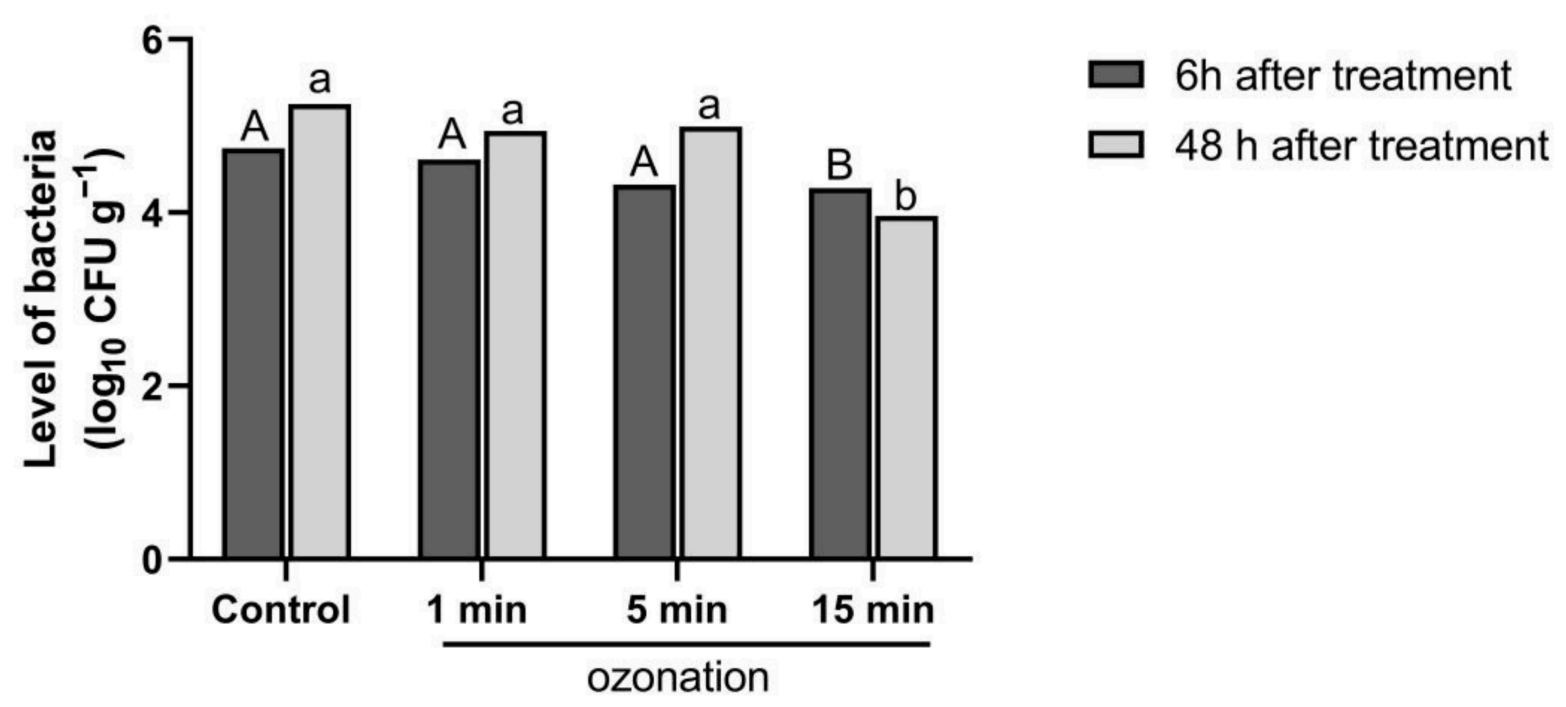
The level of bacteria in ozonated and control (zero time) meat samples after 6 and 48 h of storage in cold conditions. Mean values (*n* = 6) marked with the same lowercase or uppercase letter are not statistically significant in relation to each other for the corresponding storage time according to Tukey’s test at α = 0.05.

The effect of ozone disinfection of muscle tissue depends on different ozone concentrations, as well as on the method of its application [33]. In a study by Matłok et al. [22] conducted on poultry meat samples exposed to gaseous ozone, it was shown that the storage time was the primary factor influencing both the number of *Enterobacteriaceae* and the total number of microorganisms on the meat surface. The applied concentration and exposure time affected the level of microbiological contamination of the raw material, but the differences were not statistically significant. Coll Cardenas et al. [15], analyzing the effect of gaseous ozone on beef samples, showed a 2.0 log_10_ cycle reduction in the total number of aerobic mesophilic heterotrophic microorganisms and a 0.7 log cycle reduction in the number of *E. coli* after 1 day at 0 °C. In turn, the use of gaseous ozone at a concentration of 154 mg/m3 at 0 or 4 °C led to a reduction of only 0.5 log cycles in the number of aerobic mesophilic heterotrophic microorganisms and a 0.6–1.0 log cycle reduction in the number of *E. coli* after a 3 h exposure time. This indicates that ozonation of beef samples combined with their simultaneous storage under refrigerated conditions improved the reduction in CFU and thus is a method that allows for extending the shelf life of the tested meat samples. The studies by Cho et al. [26] also indicate a reduction in the number of *E. coli* by 0.53 log CFU/g after exposing ground beef to 10 mg O_3_/h for one day at 4 °C. The bacteriostatic effect of ozone is also confirmed by other research results [24,25,35,42,43,44]. Figure 3 and Figure 4 present the levels of bacteria, yeasts, and molds in horse meat samples after 6 and 48 h of refrigerated storage.

In the studies conducted in this work, the application of ozone for 5 and 15 min resulted in a significant reduction in the number of yeasts and molds in horse meat samples compared to the control samples and samples subjected to ozonation for 1 min (*p* < 0.05). The lowest levels of yeast and molds, at 3.24 and 3.04 log_10_ CFU/g, were observed after 15 min of ozonation in both time intervals, while the initial presence of microflora in the control samples was 3.72 and 4.74 log_10_ CFU/g, respectively. Importantly, this trend was observed after both 6 and 48 h of refrigerated storage. The use of gaseous ozone for 5 and 15 min also led to a reduction in the total bacterial count in the tested horse meat samples compared to the control samples and those treated with 1 min ozonation, but only after 6 h of refrigerated storage. After 48 h of storage of the tested horse meat samples, the use of gaseous ozone for 15 min significantly reduced the total bacterial count compared to the other experimental groups (*p* < 0.05). During the analyzed time period, the overall bacterial contamination was reduced from 5.25 log_10_ CFU/g in control samples to 3.96 log_10_ CFU/g in horse meat samples ozonated for 15 min. This distribution of test results was expected, as ozone is an extremely strong oxidant (with a reduction potential of 2.07 V), making it an effective tool for meat tissue disinfection. The mechanism by which ozone destroys microbiological contaminants is based on the direct oxidation and disintegration of microbial cellular structures, ultimately leading to their death. Ozone primarily damages components of the bacterial cell wall and cytoplasmic membrane. It also oxidizes unsaturated fatty acids and lipids, which leads to increased membrane permeability. As a result of membrane damage, so-called cell lysis (disintegration) occurs, i.e., its disruption. The cell’s interior (protoplasm) leaks out, immediately killing the microorganism. Ozone also penetrates cells and oxidizes key enzymes, proteins, and nucleic acids (DNA/RNA). Destruction of these components prevents bacteria and molds from functioning and reproducing. Furthermore, as ozone decomposes in water in muscle tissue, it releases free radicals (such as the hydroxyl radical), which are even more reactive than ozone itself, accelerating the process of pathogen destruction [8,45,46].

## 3. Materials and Methods

### 3.1. Experimental Design

The research material consisted of *longissimus thoracis* (LT) muscle collected from six right horse carcasses. The selection of carcasses used in the study was based on purchase documentation, and the individuals involved in the experiment had no contact with live animals. Slaughter was carried out in accordance with procedures commonly applied in the meat industry and in compliance with European Union regulations [47]. Animals for the study were randomly selected. Before slaughter, the horses were in good physical condition, approximately 12 years old (11 ± 0.5 years), and weighed between 500 and 540 kg (520 ± 30 kg). The age of the horses was determined based on purchase documentation. The experimental material included samples collected from two horse breeds, namely Malopolski and Silesian. Among the animals, 50% were geldings and 50% were mares. After transport, horses were held for approximately 24 h in separate pens within animal holding facilities. Prior to slaughter, the animals were stunned using a captive bolt pistol. Following slaughter and post-mortem carcass assessment, the carcass was chilled under standardized slaughterhouse conditions at 0–2 °C, 90–95% relative humidity, and an air velocity of 1.5–2.0 m/s for the first 24 h, until the internal muscle temperature decreased below 7 °C. After chilling, the carcass was deboned, and the LT muscle was divided into individual portions. In order to assess the effect of ozone treatment on horsemeat quality, meat portions weighing 3000 g were collected from the *longissimus thoracis* muscle at the level of the 13th–16th thoracic vertebrae. Additionally, a 1000 g portion was collected from each half-carcass to serve as a control group, which did not undergo the ozonation process. Thus, the meat portions used in the experiment were obtained from a single carcass. Subsequently, the collected horse meat portions were vacuum-packed using a vacuum packer (Inauen, Schwanden, Switzerland) in high-barrier polyamide/polyethylene (PA/PE) film bags and subjected to wet aging under vacuum conditions for 7 days at 4 ± 0.5 °C. After this time, the samples were transferred to the laboratory for analysis. In practice, horse meat requires a longer maturation period compared to other meats. Therefore, all laboratory analyses were performed 7 days after slaughter (post mortem). After the wet-aging period, the packages were opened, and the meat portions were subjected to ozone treatment, with the exception of the control sample. Thus, not all the samples were stored aerobically. Following ozonation, the meat portions were stored under the experimental conditions until analysis. After opening the package, the control meat was stored under the same experimental conditions as the ozone-treated samples until analysis. Prior to laboratory analyses, a control meat sample weighing 1000 g was divided into two 500 g portions; one was analyzed after 6 h of refrigerated storage, and the other after 48 h of refrigerated storage (4 ± 0.5 °C).

### 3.2. Meat Ozonation Procedure

Each experimental treatment consisted of a 3 kg batch of *longissimus thoracis* muscle, divided into six portions of 0.5 kg each. The meat portions were placed in a plastic container on a plastic pallet and equipped with a lid (42.5 cm × 29.5 cm × 13 cm). The dimensions of each meat portion were approximately 15 cm × 8 cm × 4 cm.

For ozonation, the container was closed on three sides, after which a hose supplying ozone from the generator was inserted inside. Ozonation was performed using an L5 ozone generator (Korona Lab, Piotrków Trybunalski, Poland), with a gas flow rate of 4 L/min. Ozone concentration was monitored with a 106 M 2B Technologies sensor (Boulder, CO, USA). The ozonation process was carried out for 1, 5, and 15 min, with treatment time counted from the moment the ozone concentration inside the container reached 10 ppm. The gas supply hose was removed from the container once a concentration of 10 ppm was reached. It was reinserted when the ozone concentration decreased. For each experimental variant, a separate ozonation process was performed for the designated treatment time. Relative humidity during the ozonation process was 50 ± 5%.

After ozonation, the container was tightly sealed on all sides and stored at 4 °C (± 0.5 °C) for 6 and 48 h. After the storage process was completed, all horse meat samples were cleaned to remove external fat, connective tissue, and tendons.

### 3.3. Analytical Methods

#### 3.3.1. Chemical Composition

Moisture content was determined according to the PN-ISO 1442:2000 [48] standard, with AOAC Official Method 950.46 [49] cited as a comparable, internationally recognized equivalent for moisture determination in meat. Crude protein was determined using the Kjeldahl method according to PN-75/A-04018 [50], whereby the measured nitrogen content was converted to protein, with AOAC Official Method 981.10 cited as a comparable, internationally recognized method for crude protein determination in meat [51]. The free fat content of the samples was determined using the Soxhlet method in accordance with the PN-ISO 1444:2000 standard [52], with AOAC Official Method 960.39 cited as a comparable, internationally recognized method for fat determination in meat [53].

#### 3.3.2. The Active Acidity (pH)

The active acidity (pH) of meat was determined using an OSH 12–01 electrode and a CPC-411 pH meter (ELMETRON, Zabrze, Poland) with an accuracy of up to 0.01. The device was calibrated based on buffers with pH values 4.00 and 7.00.

#### 3.3.3. Hydration Properties

For the evaluation of forced and cooking losses, meat samples were homogenized by passing them twice through a laboratory wolf (HENDI, Warsaw, Poland) equipped with a 4.0 mm plate. The ground meat was thoroughly mixed to ensure sample homogeneity. The amount of cooking loss was determined using Janicki and Walczak’s method [54], and forced meat drip was assessed using the method of Grau and Hamm [55].

#### 3.3.4. Color Parameters and Pigment Levels

Instrumental color analysis (L*, a*, b*) was executed on the freshly cut meat surface according to the methodology outlined by Stanisławczyk et al. [56]. Measurements were performed using a Hunter Lab Ultra Scan PRO electronic spectrophoto colorimeter (Hunter Associates Laboratory, Inc., Reston, VA, USA) operating in reflectance mode. Prior to analysis, samples were allowed to bloom for 30 min at 4 °C. The device was equipped with an 8 mm aperture and operated under illuminant D65 with a 10° standard observer. Calibration was completed using a standard white tile (L*—99.18, a*—0.07, b*—0.05). The proportion of heme pigments in the meat samples was measured according to the method described by Krzywicki [57].

#### 3.3.5. Texture Parameters

The shear force of raw meat was determined using a TA texture meter. XT plus (Stable Micro System Ltd., Surrey, UK). Samples of raw meat in the shape of cylinders, cut with a 1.0 cm diameter cork borer (along muscle fibers), were cut with a Warner–Bratzler blade with a triangular notch, and the value of the force needed to cut them (N/cm^2^) was recorded. Instrumentally, the texture parameters of the meat samples were determined using Texture Profile Analysis (TPA) performed with a Texture Analyzer-CT3-25 (Brookfield, WI, USA), with a 38.1 mm diameter, 20 mm long cylindrical attachment, following the method described in a prior publication [58].

#### 3.3.6. Sensory Properties

Sensory evaluation of horse meat was carried out by a twelve-member team with proven sensory sensitivity in accordance with ISO 8586:2023 [59] and ISO 8587:2006 [60] in a laboratory organized in accordance with ISO 8589 [61]. Sensory evaluation was conducted by a selected panel of 12 assessors who had previous training and experience in the sensory assessment of meat and meat products. The assessors were selected on the basis of their demonstrated sensory sensitivity and ability to recognize and discriminate the evaluated attributes, following the general recommendations of ISO 8586:2023 [59]. Before the experimental sessions, the panel participated in an orientation session during which the definitions of the evaluated attributes, scale anchors, sample presentation procedure, and evaluation protocol were reviewed. Horse meat portions (100 g) were cooked at 95 °C until reaching an internal core temperature of 80 ± 2 °C, monitored via a digital needle-probe thermometer (Sous Vide Thermapen, MERA, Warsaw, Poland). For sensory characterization, the thermally processed samples were cooled to 20 ± 2 °C and sectioned into 1.5 cm thick slices perpendicular to the longitudinal direction of the muscle fibers. The specimens were then transferred into lidded, disposable plastic containers. Each sample was individually coded and presented to the assessors in a randomized order. All evaluations were performed in three replicates, and each evaluator assessed every experimental variant. Qualitative characteristics of the samples were appraised using a 5-point scale with the following parameters: odor intensity, flavor intensity, odor desirability, flavor desirability, juiciness, and tenderness as outlined by Stanisławczyk et al. [62]. Detailed definitions of each rating level for odor intensity, odor desirability, flavor intensity, flavor desirability, juiciness, and tenderness are presented in Table 8.

Each panelist had experience in assessing the sensory characteristics of meat and meat products. Sensory evaluation was conducted during the day at room temperature, in individual booths with white light. Samples for sensory evaluation were randomly selected. Before testing each sample, panelists took a 30 s break, during which they rinsed their mouths with mineral water.

#### 3.3.7. Thiol Group Content in Soluble Protein

One gram of tissue was homogenized in 5 mL of 100 mM Tris-HCl buffer (pH 6.8) containing 0.9% NaCl, 0.1% Triton X-100, and 2 mM EDTA-2Na. The homogenate was centrifuged at 10,000× *g* for 30 min (4 °C), and the supernatant was used for protein isolation. Subsequently, 8 mL of acetone pre-cooled to −20 °C was added to 2 mL of the supernatant and incubated at −20 °C for 16 h. After incubation, the protein precipitate was centrifuged (10,000× *g*, 4 °C, 5 min), the supernatant was discarded, and the precipitate was air-dried under a fume hood for 30 min. The precipitate was then resuspended in 0.5 mL of 100 mM Tris-HCl buffer (pH 6.8) and sonicated on ice for 10 s. Total protein concentration was determined using the Bradford method according to protocol [63], while thiol group content was measured using the Ellman reagent method as described in publication [64]. Thiol group content was expressed as glutathione equivalents (GSHE) per 1 g of total protein.

#### 3.3.8. TBARS Content Analysis

One gram of the milled sample was homogenized with 2 mL of 10% trichloroacetic acid and centrifuged at 7500× *g* for 30 min. Then, 0.5 mL of the supernatant was mixed with 0.5 mL of 0.1% thiobarbituric acid and heated at 80 °C for 20 min. After cooling, 100 µL of the reaction mixture was transferred to the wells of a 96-well plate, and absorbance was measured at 532 nm [65]. TBARS values were expressed as malondialdehyde (MDA) equivalents, calculated using the molar absorption coefficient of MDA (155 mM^−1^·cm^−1^).

#### 3.3.9. Volatile Compounds Profile Analysis Using SPME-GC-MS

A 5 g meat sample was placed in a 100 mL Erlenmeyer flask and sealed with aluminum foil. Volatile compounds were adsorbed onto a 100 μm polydimethylsiloxane (PDMS) fiber (Bellefonte, PA, USA) for 30 min at 40 °C. The SPME fiber was then inserted into the injector of the GC–MS system, where analyte desorption was carried out for 5 min at 250 °C. Separation was performed using a Varian 450 GC (Palo Alto, CA, USA) gas chromatograph coupled with a Varian 240 MS (Varian 450; Palo Alto, CA, USA), mass spectrometer. Analytes were separated on a 30 m × 0.23 mm capillary column coated with a moderately polar HP-5 stationary phase (methyl phenyl polysiloxane) with a film thickness of 0.25 μm. The oven temperature program was as follows: initial temperature of 50 °C held for 5 min, followed by an increase at a rate of 10 °C/min up to 300 °C, with a final isothermal hold for 5 min. The total analysis time was 35 min. Helium was used as the carrier gas at a flow rate of 1 mL/min, and the injector temperature was maintained at 250 °C. Electron ionization (EI) at 70 eV was performed in full-scan mode over the mass range of 5–500 *m*/*z*. Identification of analytes was achieved by comparing the obtained mass spectra with reference spectra from the NIST 08 library (Supplementary Materials S1). Kovats retention indices (*RI*) were calculated following the preliminary analysis of a C7–C30 n-alkane mixture (Merck, Darmstadt, Germany), using the following formula:

$$RI=100n+100\frac{t_{r\left(X\right)}^{\prime }-t_{r\left(n\right)}^{\prime }}{t_{r\left(n\right)}^{\prime }-t_{r\left(n+1\right)}^{\prime }}$$

where

*t*′*_r_*_(*x*)_—reduced retention time of the test substance; *t*′*_r_*_(*n*)_—reduced retention time of an alkane with *n* carbon atoms; *t*′*_r_*_(*n* + 1)_—reduced retention time of an alkane with *n* + 1 carbon atoms.

#### 3.3.10. Fatty Acid Profile Analysis

A frozen and ground meat sample (2 g; −67 °C) was homogenized in 25 mL of a methylene chloride–methanol solution (2:1, *v*/*v*). The homogenate was centrifuged at 10,000× *g* for 20 min. Subsequently, 20 mL of the supernatant was purified with 5 mL of 0.58% NaCl solution and centrifuged again at 5000× *g* for 5 min. An aliquot of 4 mL of the organic layer was evaporated to dryness under a nitrogen stream at 60 °C and saponified for 20 min at 80 °C with 4 mL of 4% NaOH in methanol. Fatty acid esterification was then performed using 3 mL of a 15% boron trifluoride solution in methanol. The resulting fatty acid methyl esters (FAMEs) were extracted with 2 mL of hexane and analyzed using a Varian 450 GC gas chromatograph coupled with a Varian 240 MS mass spectrometer. The same column, separation, and detection conditions as described in Section 3.3.9 were applied, except that the injection volume was 1 μL with a split ratio of 1:50. Fatty acid methyl esters were identified by comparing the obtained mass spectra with reference spectra from the NIST database and by analysis of a PUFA methyl ester standard (cat. no. 47085-U, Merck, Darmstadt, Germany). Fatty acid content was expressed as the percentage of the peak area of each compound relative to the total peak area of all identified fatty acids [66].

#### 3.3.11. Microbial Analysis

For microbial determination, ten grams of each meat sample were aseptically excised, transferred into a sterile stomacher bag, blended with 90 mL of sterile physiological saline solution (0.9% NaCl, *w*/*v*) and homogenized for 2 min in a BagMixer 400 stomacher (Interscience, Saint-Nom-la-Bretêche, France). Then, subsequent decimal dilutions were prepared and plated on appropriate culture media. Total aerobic counts were determined on Plate Count Agar (PCA) at 30 °C for 72 h. For yeast and mold determination, YCG agar with chloramphenicol was used, and samples were incubated at 25 °C for 5 days. The presence of *E. coli* was analyzed on TBX chromogenic medium by incubating the samples for 24 h at 44 °C. For the determination of *Bacillus cereus*, MYP agar was used. For *Salmonella* detection, SS (*Salmonella Shigella*) agar was used, and samples were incubated for 24 h at 37 °C. For the detection of *Clostridium perfringens*, TSC agar was used, and probes were incubated anaerobically at 37 °C for 24 h. Anaerobiosis was created with the Anaerocult system (Merck Millipore, Poznań, Poland). All media and supplements were purchased from Biomaxima (Lublin, Poland). After the incubation period, microbial colonies were counted, and results were expressed as log_10_ CFU/g of meat sample.

### 3.4. Statistical Analysis

Statistical analysis was performed using the Statistica 13.3 PL package from TIBCO Software Inc. (Palo Alto, CA, USA). The effect of ozonation time and refrigerated storage time on horse meat quality was evaluated using a two-way analysis of variance (ANOVA) with the GLM procedure in Statistica. All meat samples used in the experiment were obtained from the same carcass, and the *longissimus thoracis* muscle was divided into portions assigned to the different ozonation treatments. Thus, the effect of ozonation was evaluated using meat portions originating from the same carcass, minimizing variability associated with differences between carcasses. When statistical significance was observed (*p* < 0.05), means were compared using Tukey’s honestly significant difference test. All analyses were performed two or three times for each experimental repetition (*n* = 6 or *n* = 9), and the results were statistically analyzed after grouping. Table 1, Table 2, Table 3, Table 4, Table 5, Table 6 and Table 7 present the mean values and standard deviations (SD) for the individual meat quality traits.

## 4. Conclusions

This study provides a detailed assessment of the impact of gaseous ozone processing on the quality parameters and physicochemical stability of horse meat. Gaseous ozone treatment at 10 ppm reduced microbial counts in horse meat, with the greatest reduction observed after 15 min of exposure. However, this treatment was also associated with increased lipid and protein oxidation, deterioration of hydration properties, changes in color and texture, and lower tenderness scores in sensory evaluation. Among the conditions examined, exposure for 1–5 min provided a more acceptable balance between microbial reduction and preservation of the physicochemical, textural, and sensory quality of the meat. Nevertheless, this exposure range should be regarded as a practical recommendation based on the present results rather than as a fully optimized treatment.

The interpretation of these findings is limited by the use of six carcasses from animals of a relatively uniform age, a single ozone concentration, and a maximum refrigerated storage period of 48 h. Therefore, the results demonstrate a short-term reduction in microbial counts but do not provide sufficient evidence to confirm an extension of shelf life. Further studies should include different ozone concentrations, longer refrigerated storage periods, and more diverse biological material. The combined use of short ozone treatments with modified-atmosphere packaging or natural antioxidants should also be investigated to improve microbial control while limiting undesirable oxidative changes.

## Figures and Tables

**Table 1:** Chemical composition of horse meat (%, $\bar{x}\;\pm \;\mathrm{SD}$).

Specification	Control	Ozonation Time 6 h After Ozonation			Control	Ozonation Time 48 h After Ozonation			ANO- VA
		1 min	5 min	15 min		1 min	5 min	15 min	
Fat	6.60 ^ax^ ± 0.40	6.07 ± 0.10	5.77 ^b^ ± 0.42	6.10 ± 0.65	6.40 ^a^ ± 0.10	5.17 ^by^ ± 0.12	5.03 ^by^ ± 0.41	5.57 ± 0.05	T; S
Water	72.57 ± 0.32	72.60 ± 0.89	72.67 ^x^ ± 0.21	72.53 ± 0.84	71.83 ^a^ ± 0.06	71.20 ^by^ ± 0.10	71.37 ± 0.64	71.37 ± 0.11	T; S
Protein	19.60 ± 0.56	19.83 ± 0.40	20.23 ± 0.49	20.00 ± 0.52	19.60 ± 0.10	20.07 ± 0.06	20.27 ± 0.38	20.00 ± 0.10	

^a,b^—values marked with different letters in the columns differ statistically significantly between the duration of the ozonation process—*p* < 0.05. ^x,y^—values marked with different letters in the columns differ statistically significantly between the duration of the cold storage—*p* < 0.05. ANOVA: two-way analysis of variance for ozonation time T; storage in cold conditions S.

**Table 2:** pH and hydration properties of horse meat ($\bar{x}$ ± SD).

Specification	Control	Ozonation Time 6 h After Ozonation			Control	Ozonation Time 48 h After Ozonation			ANO- VA
	0 min	1 min	5 min	15 min	0 min.	1 min	5 min	15 min	
pH	5.62 ^ax^ ± 0.03	5.68 ^abxy^ ± 0.04	5.76 ^b^ ± 0.03	5.72 ^abxy^ ± 0.06	5.94 ^y^ ± 0.03	5.97 ^y^ ± 0.05	5.97 ^y^ ± 0.03	5.98 ^y^ ± 0.01	T; S; T × S
Forced drip (cm^2^)	2.17 ^ax^ ± 0.29	2.70 ^b^ ± 0.25	3.07 ^c^ ± 0.70	3.30 ^c^ ± 0.55	2.90 ^a^ ± 0.42	3.63 ^b^ ± 0.56	3.77 ^by^ ± 0.76	3.57 ^b^ ± 0.35	T; S
Cooking loss (%)	22.13 ^ax^ ± 1.88	23.82 ^a^ ± 1.38	23.84 ^a^ ± 0.41	24.43 ^b^ ± 1.36	22.94 ^a^ ± 0.88	22.56 ^a^ ± 3.19	24.67 ^b^ ± 1.80	25.84 ^by^ ± 1.09	T; S

^a,b,c^—values marked with different letters in the columns differ statistically significantly between the duration of the ozonation process—*p* < 0.05. ^x,y^—values marked with different letters in the columns differ statistically significantly between the duration of the cold storage—*p* < 0.05. ANOVA: two-way analysis of variance for ozonation time T; storage in cold conditions S.

**Table 3:** Color parameters and pigment levels in horse meat ($\bar{x}$ ± SD).

**Specification**	**Control**	**Ozonation Time** **6 h After Ozonation**			**Control**	**Ozonation Time** **48 h After Ozonation**			**ANO-** **VA**
	**0 min**	**1 min**	**5 min**	**15 min**	**0 min**	**1 min**	**5 min**	**15 min**	
L*	31.23 ^a^ ± 3.93	31.61 ^a^ ± 2.32	31.75 ^a^ ± 1.99	34.32 ^bx^ ± 3.53	30.36 ^ay^ ± 1.18	31.55 ^a^ ± 1.59	31.72 ^a^ ± 1.58	33.45 ^b^ ± 2.15	T; S;
a*	22.04 ^ax^ ± 1.89	20.19 ^a^ ± 2.26	19.70 ^b^ ± 1.53	18.18 ^by^ ± 2.05	20.80 ^a^ ± 1.43	19.39 ^b^ ± 1.67	18.93 ^by^ ± 1.21	18.30 ^by^ ± 1.71	T; S;
b*	4.57 ^a^ ± 1.24	5.33 ^b^ ± 1.39	5.74 ^bx^ ± 1.14	6.98 ^cx^ ± 0.70	3.93 ^ay^ ± 1.17	4.63 ^b^ ± 1.20	4.82 ^b^ ± 0.72	5.69 ^cx^ ± 1.18	T; S;
Mb (%)	25.27 ^ax^ ± 2.93	21.08 ^b^ ± 3.59	21.03 ^b^ ± 4.22	17.64 ^cy^ ± 1.87	24.62 ^ax^ ± 4.99	23.18 ^a^ ± 3.87	23.15 ^a^ ± 0.71	15.29 ^by^ ± 3.20	T; S;
MMb (%)	24.94 ^ax^ ± 3.03	28.56 ^bx^ ± 5.26	33.93 ^a^ ± 4.90	35.85 ^ay^ ± 2.53	31.28 ^a^ ± 2.32	32.29 ^a^ ± 1.47	32.51 ^a^ ± 1.61	36.90 ^by^ ± 2.28	T; S;
Mb•O_2_ (%)	53.99 ^ax^ ± 1.90	53.81 ^ax^ ± 3.51	45.05 ^b^ ± 5.21	38.88 ^cy^ ± 2.32	52.20 ^ax^ ± 5.32	45.57 ^b^ ± 3.22	43.09 ^b^ ± 1.04	39.92 ^cy^ ± 2.17	T; S;
OZB (mg/g)	605.07 ^ax^ ± 32.38	461.28 ^b^ ± 28.17	427.08 ^cy^ ± 73.99	410.94 ^cy^ ± 65.59	587.53 ^ax^ ± 55.37	470.68 ^b^ ± 24.36	429.54 ^cy^ ± 44.92	428.30 ^cy^ ± 46.65	T; S; T × S

^a,b,c^—values marked with different letters in the columns differ statistically significantly between the duration of the ozonation process—*p* < 0.05. ^x,y^—values marked with different letters in the columns differ statistically significantly between the duration of the cold storage—*p* < 0.05. ANOVA: two-way analysis of variance among ozonation time T; storage in cold conditions S. L*—lightness; a*—redness; b*—yellowness; Mb—myoglobin; MMb—metmyoglobin; Mb•O_2_—oxymyoglobin; OZB—total heme pigment content.

**Table 4:** Parameters textures of horse meat ($\bar{x}$ ± SD).

Specification	Control	Ozonation Time 6 h After Ozonation			Control	Ozonation Time 48 h After Ozonation			ANO-VA
	0 min	1 min	5 min	15 min	0 min	1 min	5 min	15 min	
Shear force (N/cm^2^)	67.39 ^a^ ± 1.10	54.93 ^b^ ± 0.78	66.02 ^a^ ± 1.30	91.53 ^cy^ ± 1.84	53.27 ^a^ ± 2.48	39.24 ^bx^ ± 0.46	43.46 ^bx^ ± 0.90	63.47 ^c^ ± 1.60	T; S; T × S
Hardness 1 (N)	165.75 ^a^ ± 9.57	164.41 ^a^ ± 7.43	139.26 ^b^ ± 6.46	216.45 ^cx^ ± 6.24	160.22 ^a^ ± 5.23	122.89 ^by^ ± 3.56	122.80 ^by^ ± 5.45	205.36 ^cy^ ± 6.06	T; S;
Hardness 2 (N)	113.21 ^a^ ± 3.11	112.66 ^a^ ± 4.69	80.19 ^bx^ ± 3.01	137.28 ^cy^ ± 4.27	146.13 ^ay^ ± 6.77	79.50 ^by^ ± 3.94	99.98 ^b^ ± 7.78	118.40 ^c^ ± 4.31	T; S;
Stiffness 5 (N)	23.10 ^a^ ± 2.27	40.34 ^b^ ± 2.66	16.32 ^ax^ ± 6.62	28.79 ^c^ ± 2.12	53.20 ^a^ ± 2.37	16.09 ^bx^ ± 2.58	14.63 ^bx^ ± 2.38	61.06 ^cy^ ± 3.81	T; S; T × S
Stiffness 8 (N)	135.49 ^ax^ ± 8,94	108.33 ^b^ ± 7.50	94.47 ^b^ ± 3.62	123.85 ^a^ ± 3.18	149.91 ^ax^ ± 4.30	82.63 ^b^ ± 3.68	68.35 ^cy^ ± 2.75	152.21 ^ax^ ± 5.99	T; S; T × S
Adhesiveness (mJ)	2.57 ^acy^ ± 0.49	2.73 ^cy^ ± 0.45	2.73 ^cy^ ± 0.49	1.43 ^bxy^ ± 0.27	1.33 ^axy^ ± 0.08	1.97 ^cxy^ ± 0.10	2.80 ^by^ ± 0.33	1.03 ^ax^ ± 0.15	T; S;
Cohesiveness	0.25 ^a^ ± 0.04	0.18 ^b^ ± 0.05	0.13 ^bx^ ± 0.05	0.31 ^ay^ ± 0.09	0.26 ^a^ ± 0.03	0.22 ^a^ ± 0.04	0.12 ^bx^ ± 0.05	0.32 ^cy^ ± 0.07	T; S; T × S
Elasticity (mm)	3.80 ^a^ ± 0.96	4.26 ^b^ ± 0.51	3.12 ^cx^ ± 0.83	5.19 ^dy^ ± 0.62	4.34 ^ay^ ± 0.76	5.14 ^by^ ± 0.20	3.82 ^c^ ± 0.28	4.73 ^ay^ ± 0.30	T; S;
Resilience	0.10 ^a^ ± 0.01	0.09 ^bx^ ± 0.04	0.08 ^bx^ ± 0.04	0.13 ^a^ ± 0.06	0.11 ^a^ ± 0.02	0.10 ^a^ ± 0.03	0.08 ^ax^ ± 0.02	0.16 ^by^ ± 0.06	T; S; T × S
Chewiness (mJ)	137.23 ^ax^ ± 4.99	234.27 ^b^ ± 3.08	267.10 ^c^ ± 5.71	387.53 ^dy^ ± 8.28	122.00 ^ax^ ± 6.20	238.10 ^b^ ± 7.56	292.87 ^cy^ ± 6.82	331.17 ^dy^ ± 5.28	T; S; T × S

^a,b,c^—values marked with different letters in the columns differ statistically significantly between the duration of the ozonation process—*p* < 0.05. ^x,y^—values marked with different letters in the columns differ statistically significantly between the duration of the cold storage—*p* < 0.05. ANOVA: two-way analysis of variance for ozonation time T; storage in cold conditions S.

**Table 5:** Volatile compounds profile in ozonated and control (zero time) meat sample after 6 and 48 h of storage in cold conditions ($\bar{x}$).

No	RT	RI Calc. *	RI Ref. **	Compound Name	CAS No	MW	Peak Share in the Chromatogram [%]							
							6 h After Ozonation				48 h After Ozonation			
							Control	1 min	5 min	15 min	Control	1 min	5 min	15 min
1	8.22	960	925–996	Benzaldehyde	100-52-7	106	44.71	n.d.	n.d.	n.d.	<LOQ	<LOQ	22.89	34.81
3	18.19	1612	-	9,17-Octadecadienal	56554-35-9	264	n.d.	n.d.	n.d.	20.50	23.97	16.84	20.81	n.d.
4	19.32	1707	-	Z.E-3,13-Octadecadien-1-ol	73332-92-0	266	29.66	53.94	69.53	50.45	51.43	74.20	12.18	8.14
5	20.41	1759	-	Z.E-2,13-Octadecadien-1-ol	-	266	25.63	46.06	30,47	29.05	24.59	8.96	44.12	57.05

RT—retention time; RI calc. *—retention index calculated; RI ref. **—retention index referenced (from pherobase.com); MW—molecular weight; n.d.—not detected; <LOQ—below limit of quantification.

**Table 6:** The fatty acid composition in ozonated and control (zero time) meat samples after 6 and 48 h of storage in cold conditions ($\bar{x}$ ± SD).

No	Compound Name	Ordinary Compound Name	Fatty Acid Composition [%] Mean ± SD								
			6 h After Ozonation				48 h After Ozonation				
			Control	1 min	5 min	15 min	Control	1 min	5 min	15 min	AN-OVA
1	undecanoic acid, 10-methyl	-	0.20 ± 0.01 ^ax^	0.22 ± 0.01 ^ax^	0.24 ± 0.05 ^ax^	0.21 ± 0.01 ^ax^	0.21 ± 0.02 ^ax^	0.23 ± 0.00 ^ax^	0.21 ± 0.01 ^ax^	0.20 ± 0.02 ^ax^	
2	9-tetradecenoic acid	myristoleic acid	0.60 ± 0.01 ^ax^	0.65 ± 0.01 ^ax^	0.60 ± 0.06 ^ax^	0.57 ± 0.02 ^ax^	0.262 ± 0.00 ^ay^	0.56 ± 0.01 ^by^	0.67 ± 0.03 ^bx^	0.55 ± 0.06 ^bx^	T, S
3	tridecanoic acid, 12-methyl	isomyristic acid	4.20 ± 0.13 ^ax^	4.14 ± 0.08 ^ax^	3.98 ± 0.03 ^bx^	4.03 ± 0.04 ^bx^	4.27 ± 0.11 ^ax^	4.00 ± 0.08 ^ax^	4.13 ± 0.07 ^ax^	4.12 ± 0.25 ^ax^	T
4	tetradecanoic acid, 13-methyl	isopentadecanoic acid	0.29 ± 0.02 ^ax^	0.33 ± 0.00 ^ax^	0.29 ± 0.02 ^ax^	0.29 ± 0.01 ^ax^	0.31 ± 0.04 ^ax^	0.37 ± 0.02 ^ax^	0.27 ± 0.01 ^ax^	0.27 ± 0.02 ^ax^	
5	pentadecanoic acid, 14-methyl	isohexadecanoic acid	0.90 ± 0.00 ^ax^	1.18 ± 0.04 ^ax^	1.12 ± 0.07 ^ax^	1.02 ± 0.02 ^ax^	0.94 ± 0.08 ^ax^	1.14 ± 0.07 ^bx^	1.04 ± 0.00 ^ax^	0.95 ± 0.01 ^ax^	T
6	9-hexadecenoic acid	palmitoleic acid	11.67 ± 3.14 ^ax^	9.43 ± 0.57 ^bx^	9.26 ± 0.67 ^bx^	8.41 ± 0.15 ^bx^	8.72 ± 0.93 ^ay^	7.91 ± 0.24 ^by^	9.10 ± 0.15 ^ax^	8.67 ± 0.52 ^ax^	T, S
7	heptadecanoic acid	margaric acid	15.10 ± 1.71 ^ax^	15.31 ± 0.33 ^ax^	15.70 ± 0.25 ^ax^	15.28 ± 0.18 ^ax^	15.29 ± 0.72 ^ax^	14.05 ± 0.08 ^by^	14.92 ± 0.17 ^by^	17.44 ± 1.01 ^cy^	T, S
8	8-heptadecenoic acid	-	0.58 ± 0.10 ^ax^	0.61 ± 0.04 ^ax^	0.58 ± 0.02 ^ax^	0.52 ± 0.03 ^ax^	0.56 ± 0.06 ^ax^	0.72 ± 0.01 ^by^	0.57 ± 0.01 ^ax^	0.52 ± 0.05 ^ax^	T, S
9	9,12-octadecadienoic acid	linoleic acid	20.07 ± 0.04 ^ax^	20.65 ± 0.37 ^ax^	18.56 ± 3.55 ^ax^	21.92 ± 0.14 ^ax^	20.48 ± 0.61 ^ax^	21.96 ± 0.10 ^ay^	20.60 ± 0.26 ^ay^	14.76 ± 1.97 ^by^	T, S
10	9-octadecenoic acid	oleic acid	37.95 ± 1.19 ^ax^	40.15 ± 0.64 ^bx^	41.55 ± 2.73 ^xb^	39.35 ± 0.27 ^bx^	40.03 ± 0.98 ^ay^	41.54 ± 0.85 ^ax^	40.00 ± 0.61 ^xa^	41.93 ± 0.15 ^ax^	T, S
11	heptadecanoic acid, 16-methyl	-	4.16 ± 0.14 ^ax^	3.52 ± 0.10 ^bx^	4.03 ± 0.11 ^ax^	4.00 ± 0.04 ^ax^	4.21 ± 0.04 ^ax^	3.80 ± 0.09 ^bx^	3.90 ± 0.05 ^ax^	5.40 ± 0.12 ^cy^	T, S
12	5,8,11,14-eicosatetraenoic acid	arachidonic acid	1.75 ± 0.16 ^ax^	1.43 ± 0.03 ^bx^	1.87 ± 0.22 ^ax^	1.73 ± 0.01 ^ax^	1.83 ± 0.30 ^ax^	0.96 ± 0.30 ^by^	2.03 ± 0.05 ^ay^	2.24 ± 0.06 ^cy^	T, S
13	8,11,14-heptadecatrienoic acid	-	0.31 ± 0.03 ^ax^	0.31 ± 0.06 ^ax^	0.32 ± 0.10 ^ax^	0.28 ± 0.03 ^ax^	0.40 ± 0.01 ^ay^	0.28 ± 0.07 ^bx^	0.34 ± 0.01 ^ax^	0.39 ± 0.01 ^ay^	T, S
14	11-eicosenoic acid	gondoic acid	1.65 ± 0.11 ^ax^	1.50 ± 0.18 ^ax^	1.19 ± 0.39 ^bx^	1.74 ± 0.12 ^ax^	1.57 ± 0.35 ^ax^	2.05 ± 0.13 ^by^	1.56 ± 0.03 ^ay^	1.72 ± 0.13 ^ax^	T, S
15	4,7,10,13,16,19-docosapentaenoic acid	-	0.58 ± 0.05 ^ax^	0.54 ± 0.02 ^ax^	0.63 ± 0.06 ^bx^	0.61 ± 0.02 ^ax^	0.58 ± 0.02 ^ax^	0.44 ± 0.00 ^ay^	0.66 ± 0.04 ^ax^	0.85 ± 0.01 ^cy^	T, S
Sum of saturated fatty acid (SFA)			24.86 ^ax^	24.71 ^ax^	25.43 ^ax^	24.84 ^ax^	25.22 ^ax^	23.58 ^ax^	24.47 ^ax^	28.38 ^by^	T, S
Sum of monounsaturated fatty acid (MUFA)			52.43 ^ax^	52.35 ^ax^	53.19 ^ax^	50.61 ^bx^	51.50 ^ax^	52.78 ^ax^	51.90 ^ax^	53.38 ^ax^	T, S
Sum of polyunsaturated fatty acid (PUFA)			22.71 ^ax^	22.94 ^ax^	21.38 ^ax^	24.58 ^ax^	23.29 ^ax^	23.64 ^ax^	23.62 ^ax^	18.23 ^by^	T, S

^a,b,c^—values marked with different letters in the columns differ statistically significantly between the duration of the ozonation process—*p* < 0.05. ^x,y^—values marked with different letters in the columns differ statistically significantly between the duration of the cold storage—*p* < 0.05. ANOVA: two-way analysis of variance for ozonation time T; storage in cold conditions S.

**Table 7:** Sensory evaluation of horse meat (points) ($\bar{x}$ ± SD).

Specification	Control	Ozonation Time 6 h After Ozonation			Control	Ozonation Time 48 h After Ozonation			ANO- VA
	0 min	1 min	5 min	15 min	0 min	1 min	5 min	15 min	
Odor (intensity)	4.25 ^a^ ± 0.45	4.00 ^b^ ± 0. 15	4.00 ^b^ ± 0. 14	4.25 ^a^ ± 0.42	4.00 ^a^ ± 0. 12	4.00 ^a^ ± 0. 11	4.25 ^b^ ± 0.44	4.25 ^b^ ± 0.43	T
Odor (desirability)	4.25 ^ax^ ± 0.42	4.25 ^ax^ ± 0.45	4.00 ^b^ ± 0.01	4.00 ^b^ ± 0. 14	3.25 ^ay^ ± 0.42	3.75 ^b^ ± 0.45	4.00 ^c^ ± 0. 15	4.00 ^c^ ± 0. 14	T; S;
Juiciness	3.50 ^a^ ± 0. 15	3.75 ^b^ ± 0.40	4.25 ^cx^ ± 0.43	3.50 ^a^ ± 0.18	3.25 ^ay^ ± 0.41	4.00 ^b^ ± 0. 13	4.00 ^b^ ± 0. 13	3.25 ^ay^ ± 0.42	T; S;
Tenderness	3.75 ^a^ ± 0.20	3.70 ^b^ ± 0. 12	4.00 ^cx^ ± 0. 14	2.75 ^dy^ ± 0.20	3.25 ^a^ ± 0.40	4.00 ^bx^ ± 0. 15	4.00 ^bx^ ± 0. 14	3.00 ^c^ ± 0.17	T; S;
Flavor (intensity)	4.50 ^a^ ± 0. 12	4.25 ^b^ ± 0.43	4.00 ^c^ ± 0. 15	4.00 ^c^ ± 0. 13	4.00 ^a^ ± 0. 15	4.00 ^a^ ± 0. 14	4.25 ^b^ ± 0.41	4.25 ^b^ ± 0.44	T;
Flavor (desirability)	4.25 ^ax^ ± 0.40	4.25 ^ax^ ± 0.44	4.00 ^b^ ± 0. 13	4.50 ^cy^ ± 0. 14	3.75 ^ay^ ± 0.42	4.00 ^b^ ± 0. 12	4.00 ^b^ ± 0. 15	3.75 ^ay^ ± 0.43	T; S;

^a,b,c,d^—values marked with different letters in the columns differ statistically significantly between the duration of the ozonation process—*p* < 0.05. ^x,y^—values marked with different letters in the columns differ statistically significantly between the duration of the cold storage—*p* < 0.05. ANOVA: two-way analysis of variance for ozonation time T; storage in cold conditions S.

**Table 8:** Sensory assessment chart.

Score	Odor Intensity	Odor Desirability	Flavor Intensity	Flavor Desirability	Juiciness	Tenderness
1	Very weak or imperceptible	Highly undesirable	Very weak or imperceptible	Highly undesirable	Very dry	Very hard
2	Weak	Undesirable	Weak	Undesirable	Slightly dry	Slightly hard
3	Moderate	Neither desirable nor undesirable	Moderate	Neither desirable nor undesirable	Moderately juicy	Moderately tender
4	Strong	Desirable	Strong	Desirable	Juicy	Tender
5	Very strong	Highly desirable	Very strong	Highly desirable	Very juicy	Very tender

## Data Availability

The original contributions presented in the study are included in the article; further inquiries can be directed to the corresponding author.

## Supplementary Materials

The following supporting information can be downloaded at: https://www.mdpi.com/article/10.3390/molecules31183315/s1. Supplementary Materials S1: Mass spectra of identified volatile compounds in horse meat; Statement S2: Statement regarding sensory evaluation study.
