# Supplementary material for: The Influence of the Ozonation Process on the Quality Parameters and Physicochemical Stability of Horse Meat
Source: Molecules. 2026 Sep 18;31(18):3315. doi: 10.3390/molecules31183315 (PMC13609763; doi:10.3390/molecules31183315)
Supplement: Supplementary file 1 [file molecules-31-03315-s001.zip › molecules-4495606-Supplementary Materials/molecules-4495606-Statement S2.pdf]

Statement Regarding Sensory Evaluation Study

We, the undersigned, hereby declare that participation in the sensory studies was entirely voluntary and based on the informed consent of the participants. The food evaluation procedure conducted was non-invasive and did not involve any risk to the participants' health.

We further declare that throughout the entire process of data collection and analysis, full protection of participants' privacy and confidentiality of personal data was ensured, in accordance with applicable legal regulations and ethical standards.

Authors

Renata Stanisławczyk, Tomasz Piechowiak, Marian Gil, Dorota Grabek-Lejko, Maciej Balawejder
