# Supplementary material for: The Influence of the Ozonation Process on the Quality Parameters and Physicochemical Stability of Horse Meat
Source: Molecules. 2026 Sep 18;31(18):3315. doi: 10.3390/molecules31183315 (PMC13609763; doi:10.3390/molecules31183315)
Supplement: Supplementary file 1 [file molecules-31-03315-s001.zip › molecules-4495606-Supplementary Materials/molecules-4495606-Supplementary Materials S1.pdf]

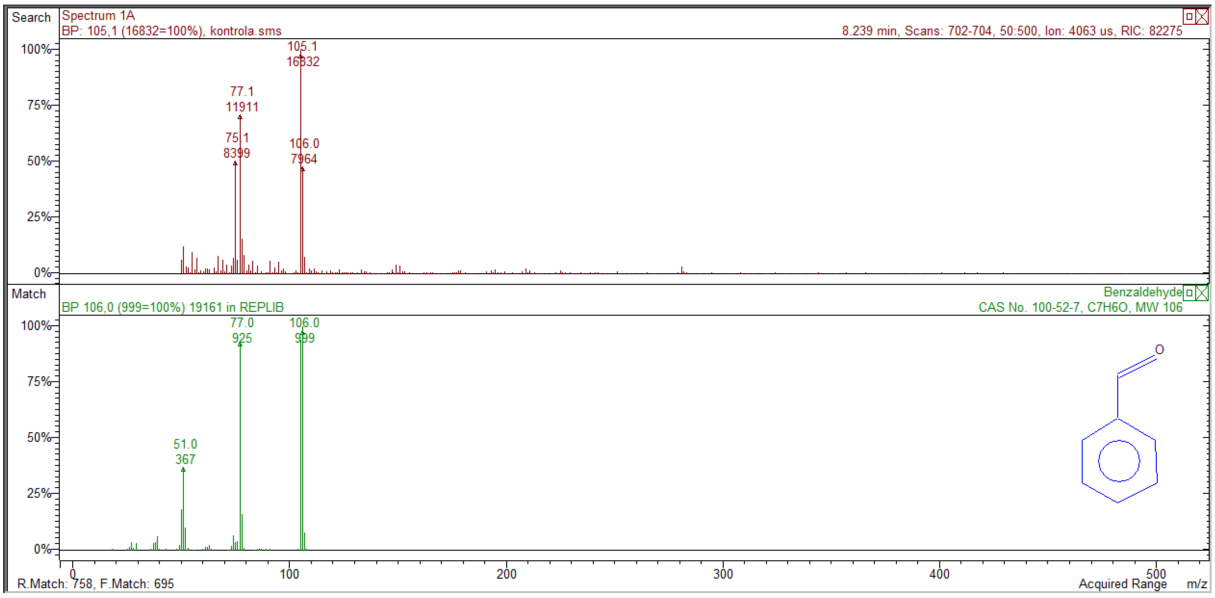
Search
Spectrum 1A
BP: 105,1 (16832=100%), kontrola.sms
8.239 min, Scans: 702-704, 50:500, Ion: 4063 us, RIC: 82275
105.1
16832
77.1
11911
75.1
8399
106.0
7964
Match
Benzaldehyde
BP 106,0 (999=100%) 19161 in REPLIB
CAS No. 100-52-7, C7H6O, MW 106
77.0
925
106.0
999
51.0
367
O
R.Match: 758, F.Match: 695
Acquired Range
m/z

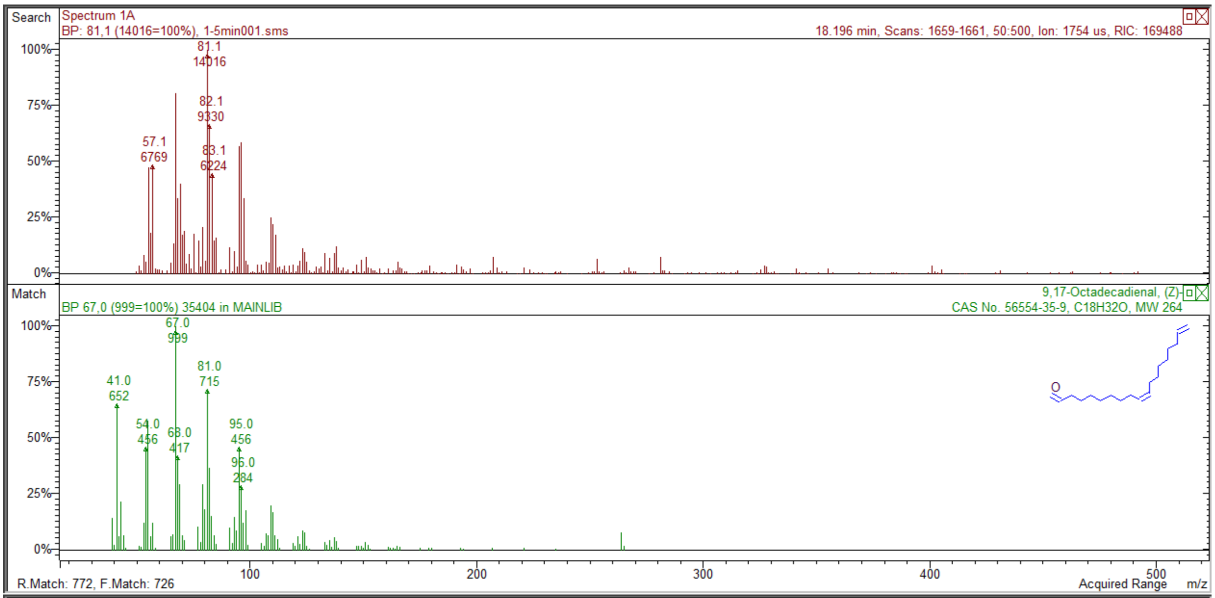
Search
Spectrum 1A
BP: 81,1 (14016=100%), 1-5min001.sms
18.196 min, Scans: 1659-1661, 50:500, Ion: 1754 us, RIC: 169488
81.1
14016
82.1
9330
57.1
6769
83.1
6224
Match
9,17-Octadecadienal, (Z)-
BP 67,0 (999=100%) 35404 in MAINLIB
CAS No. 56554-35-9, C18H32O, MW 264
67.0
999
81.0
715
41.0
652
54.0
456
68.0
417
95.0
456
96.0
284
O
R.Match: 772, F.Match: 726
Acquired Range
m/z

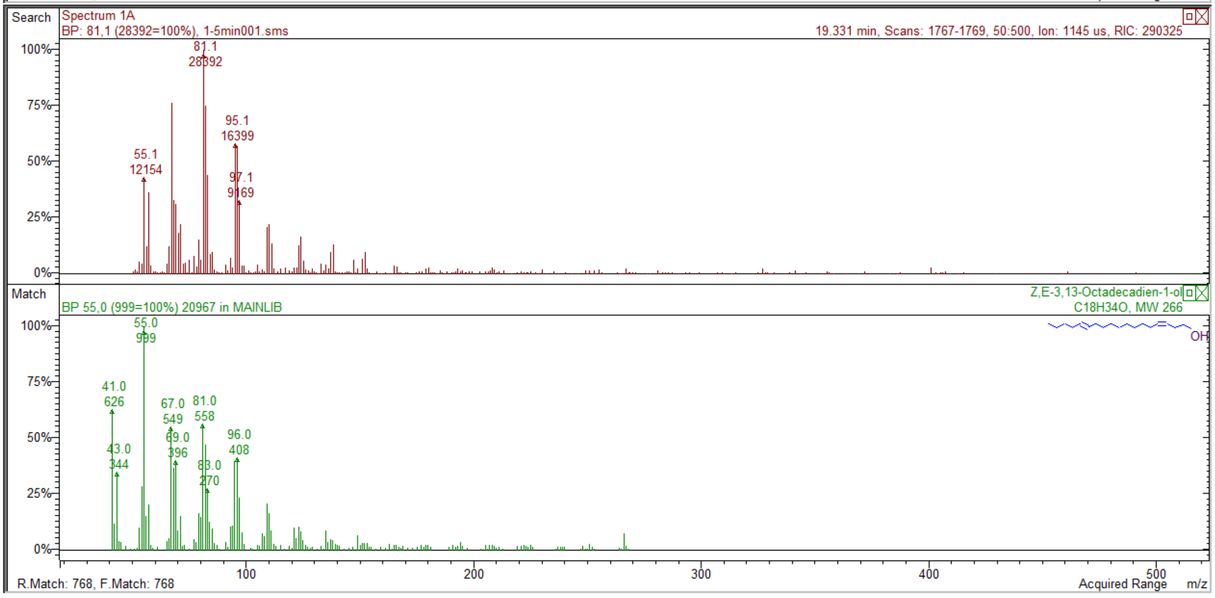
Search
Spectrum 1A
BP: 81,1 (28392=100%), 1-5min001.sms
19.331 min, Scans: 1767-1769, 50:500, Ion: 1145 us, RIC: 290325
81.1
28392
95.1
16399
55.1
12154
97.1
9169
Match
Z,E-3,13-Octadecadien-1-ol
BP 55,0 (999=100%) 20967 in MAINLIB
C18H34O, MW 266
OH
55.0
999
41.0
626
67.0
549
81.0
558
43.0
344
69.0
396
96.0
408
83.0
270
R.Match: 768, F.Match: 768
Acquired Range
m/z

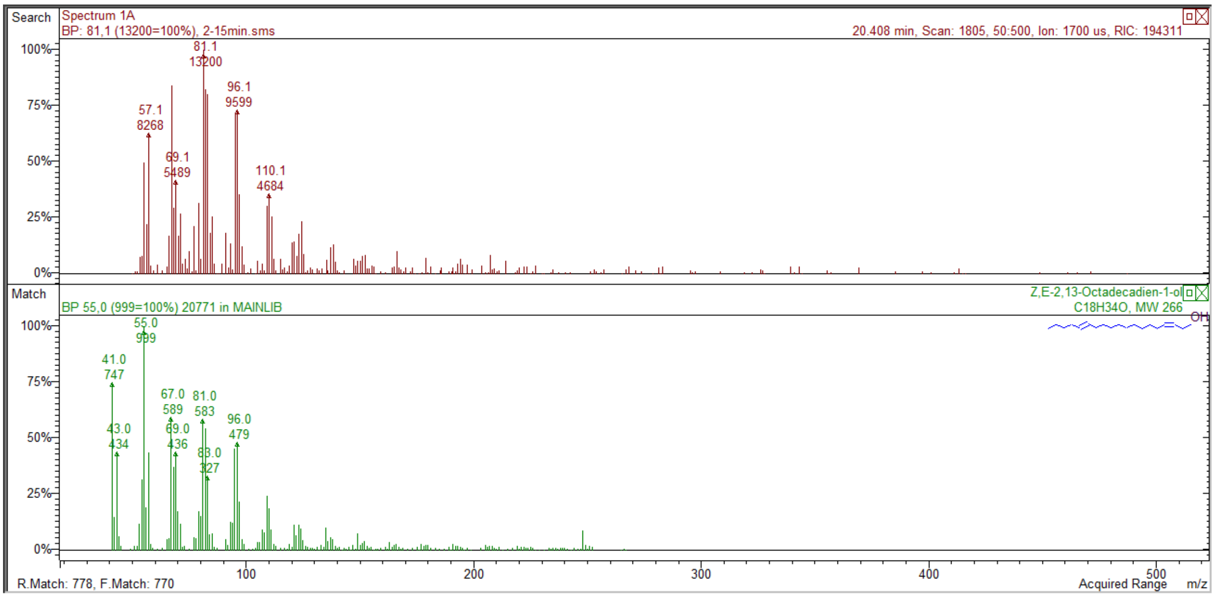
Search
Spectrum 1A
BP: 81,1 (13200=100%), 2-15min.sms
20.408 min, Scan: 1805, 50:500, Ion: 1700 us, RIC: 194311
81.1 13200
96.1 9599
57.1 8268
69.1 5489
110.1 4684
Match
Z,E-2,13-Octadecadien-1-ol
C18H34O, MW 266
BP 55,0 (999=100%) 20771 in MAINLIB
55.0 999
41.0 747
67.0 589
81.0 583
96.0 479
43.0 434
69.0 436
83.0 327
R.Match: 778, F.Match: 770
Acquired Range
m/z
